# The effects of charring on sunflower (*Helianthus annuus* L) achenes and kernels: Taphonomic and behavioral implications

**DOI:** 10.1371/journal.pone.0326159

**Published:** 2025-06-12

**Authors:** John P. Hart

**Affiliations:** Research and Collections Division, New York State Museum, Albany, New York, United States of America; Government College University Faisalabad, PAKISTAN

## Abstract

Sunflower (*Helianthus annuus* L) is one of several seed-bearing annual plants that were domesticated in eastern North America 5000–3800 years ago. When found in the archaeological record the plant’s fruits (achenes) are generally charred. Charring causes changes in the lengths and widths of achenes and their enclosed kernels. Correction factors for these changes derived from controlled heating experiments have been proposed to estimate the original size of charred achenes. The resulting corrected sizes are used to distinguish achenes from domesticated and wild sunflowers. The current study aimed to assess the accuracy of these correction factors and explore morphological differences in hulled and unhulled kernels after heating in 20°C increments from 200 to 420°C. The results indicate the proposed correction factors are inaccurate, potentially leading to type 1 and type 2 errors in assessing domestication status of achenes; the use of correction factors to identify domesticated sunflower achenes is not warranted. Rather, size comparisons of charred archaeological to charred wild contemporary sunflower achenes reported in a previous experiment are less likely to result in assignment errors. Results also indicate it is possible to distinguish between kernels that were hulled before charring from those that were not hulled based on length:width ratios and the presence of longitudinal ridges on unhulled kernels.

## Introduction

As confirmed by ancient DNA analysis, sunflower (*Helianthus annuus* L) is one of several indigenous annual plants to have been domesticated in eastern North America between 5000 and 3800 years ago [1,2]. It and squash (*Cucurbita pepo* L ssp. *ovifera*) are the only representatives of these crops to contribute to current global agricultural production. Today, globally, sunflower kernels are the fourth greatest source of vegetable cooking oil, they are consumed by humans in a wide range of culinary contexts, and the plant is used in biodiesel production [3,4].

Sunflower indehiscent fruits (achenes) form from small flowers (disc florets) within the central disc of the composite inflorescence (capitulum) [5]. The fruits consist of a thin lignified pericarp enclosing a single dicotyledonous kernel with a thin seed coat. Hundreds to over 1000 achenes are produced in the disc depending on variety [6,7].

Most finds of sunflower achenes and kernels on open-air sites in eastern North America are charred. Paleoethnobotanists use achene size to distinguish between domesticated and wild forms, an important distinction in investigations of early agriculture. Larger achenes (≥7 mm in length, > 23 length x width product) are assumed to be from domesticated plants. Exposure to high heat and consequent charring can cause pericarps and kernels to shrink through the loss of water and oil. Therefore, as reviewed in the next section, several charring experiments have been performed by paleoethnobotanists to understand the degree of shrinkage in these structures and derive correction factors for achenes and kernels to determine the likely original size of achenes, enabling analysts to distinguish small early domesticated achenes and kernels from those of wild plants [2,8–13].

Carbonized kernels from possible domesticated plants are present at the Napolean Hollow site in present-day Illinois dating as early as 5800 years ago [2]. Carbonized kernels thought to be from domesticated sunflower occur as early as 4800 years ago at the Hayes site in present-day Tennessee [2]. After its domestication, there was a gradual increase in the size of sunflower achenes reaching their largest pre-contact size in eastern North America after 1100−1000 years ago in the riverine interior [13]. Regional variation in achene size manifests with, for example, larger achenes to the south and smaller achenes to the north at Iroquoian sites in Ontario [14] and smaller achenes in southern New England compared to those from contemporaneous riverine interior sites [15].

Despite the crop’s long history and its mentions in ethnohistorical accounts, sunflower achenes and kernels generally occur in small numbers, if at all, in eastern North American open-air site charred macrobotanical assemblages. Finds of undreds to thousands of achenes or kernels at a given site are rare occurrences [e.g., 14,16–19]. It has been suggested that this is because achenes and their components do not tolerate high temperatures well [11] and the consequent fragility of pericarps after charring [19, p. 199]. Charring experiments of achenes and hulled kernels have focused on shrinkage, changes in chemistry, and the identification of charring temperatures [e.g., 8,11]. Here, following recent charring experiments with maize (*Zea mays* L ssp. *mays*) kernels [20], common bean (*Phaseolus vulgaris* L) seeds [21], and squash (*C. pepo* ssp. *ovifera*, *C. moschata*, and *C. maxima*) seeds [22], I relate the results of controlled charring experiments on sunflower achenes and hulled and unhulled kernels. The current experiments included testing achenes and kernels for compression strength following heating in oxygen-limited conditions in series of 12 20°C increments from 200 to 420°C. These experiments were designed to determine (1) changes in achene and kernel morphologies at systematic heating intervals, (2) how compression strength compares to charred propagules of common bean and squash, and (3) the accuracy of proposed correction factors for achene shrinkage.

## Previous experiments

Several heating experiments of sunflower achenes and kernels have been previously reported. A primary focus for these experiments was determining size correction factors for charred achenes and kernels recovered from archaeological sites. The first was by Heiser in an unpublished 1953 manuscript as related by Smith [2]. In this experiment, Heiser charred 10 achenes from each of seven sunflower varieties at 370°C for 3 hours. This resulted in an average shrinkage in length of 10%, width 21%, and thickness 15%. No indication is provided as to whether the achenes were charred in aerobic or anaerobic conditions [2]. Yarnell [13] later suggested an average correction factor for charred achenes of 11% for length and 27% for width and 30% and 45% for kernels, respectively to estimate original achene size. Fecteau [23, p. 230] heated 100 sunflower achenes wrapped in aluminum foil for 15 minutes at 300°C. He noted mean decreases in length and width of 18% and 23%, respectively and an increase in thickness of 3.2%.

Wright [10,11] later ran a series of experiments with achenes buried in sand and heated at 100°C intervals from 100°C to 500°C for 5 or 50 minutes. Dried achenes carbonized when heated at 300°C for 50 minutes. Wright noted average shrinkage in dried achenes <5% for length, 25% for width, and 18% for thickness. Based on a subsequent series of experiments with heating at 60 minutes done at 160, 220, 250, 310, 340, 370, 400, 440, 500, and 600°C [8,9,12], Wright [12] suggested that the range of temperatures that dried sunflower achenes were likely to carbonize and preserve in the archaeological record was 370–500°C under completely anaerobic conditions at 50 minutes. This was based on changes on chemical and physical properties of achenes. The pericarps of fresh achenes were likely to crack as the seed expanded at temperatures between 200 and 300°C. Proteins and lipids in dried achenes survived temperatures below 310°C and so achenes were likely to decay over time. Braadbaart and Wright [8, p. 60] suggested ranges of correction factors for length (1.03–1.29) and width (1.11–1.41) based on what they considered a likely range of carbonization temperatures for achenes (310–600°C at 60 minutes). They suggested that at temperatures below 310°C the preservation of sugars and proteins, will result in microbial attack in soils, lessening chances of preservation. More recently, Smith [2] charred 100 achenes, 25 from the inflorescences of each of four wild sunflower plants at 400°C for 2 hours in anaerobic conditions. He recorded decreases in length of 2–12% and width of 4–26%. Smith also carbonized 25 kernels using the same protocols and recorded decreases in length of 10–15%. He suggested that carbonized achenes and kernels recovered from the archaeological record be compared to his results; those larger than the carbonized wild kernels or achenes can be determined to have been from domesticated sunflowers.

## Materials and methods

The aim of the present experiments was to replicate previous experiments as reviewed above, but following protocols in my earlier experiments charring maize kernels, common bean seeds, and squash seeds [20–22] following Charles and associates [24]. No permits were required for the described study, which complied with all relevant regulations. Contemporary dried achenes from two heirloom sunflower varieties, Arikara and Seneca, obtained from commercial seed-stock sources were used. Randomly selected sets of 10 achenes from each variety were heated separately at one of 12 20°C temperature increments, starting at 200°C and ending at 420°C. The temperature range was selected given what is known about temperatures that occur in soils below open wood fires [25–27] and previous heating experiments with maize kernels and common bean and squash seeds. An additional heating experiment was conducted with Arikara achenes at 440°C to assess seed survival following results at 420°C where most pericarps were partially or entirely consumed but some kernels survived (see Results).

Prior to heating each achene was weighed to the nearest 0.0001 g on an electronic balance and then measured for length, width, and thickness with an electronic digital caliper to the nearest 0.01 mm. Length was measured at the greatest distance between the distal (wide) and proximal (pointed) ends, width at the greatest lateral distance, and thickness at the widest distance between the two faces. These latter two measurements were typically taken at or near the distal end of the achene.

To affect oxygen-limited conditions, each achene was separately wrapped loosely in aluminum foil to allow expansion, and placed in a 10 ml ceramic crucible, which was then filled with sand. The 10 crucibles were placed on a tray in the center of a pre-heated Lindberg Platinell II type KP2 box furnace and heated for 2 hours. At the end of 2 hours, the crucibles were removed from the furnace, the sand was dumped, the achenes were unwrapped and remeasured. The compression strength of each achene was measured in newtons (N) with a digital Willspring GWJ–2 grain hardness tester (accuracy = ±2%). The achenes were laid flat on the platform and force was applied with the indenter until an audible and/or visible crack occurred. Achenes that were completely consumed or the pericarps partially or wholly consumed at 400°C and 420°C were scored as 0 N. Once cracked, achenes were opened to determine if the kernels remained intact and, if so, they were measured for length, width, and thickness in the same manner as the achenes. In some instances, the kernels were broken during compression testing of the achene or broke or crumbed when being extracted and could not be measured. In cases where 5 or more kernels of either variety could not be measured for a given temperature, 10 additional achenes of the variety were heated at that temperature for 2 hours. These achenes were measured for all metrics pre- and post-heating but were not subjected to compression testing (S1 Table). The kernels were then extracted and those that survived extraction intact were measured. To assess the effects of heating on hulled kernels, separate random sets of 10 achenes from both varieties were measured. Each achene was then cut at the edges using a razor blade to separate the pericarp into approximately equal halves. The kernels were extracted measured, heated, and remeasured using the same protocols as the achenes. Any kernel damaged during extraction was discarded and a new achene was randomly selected for kernel extraction. Most often when a kernel was damaged it had split into cotyledons when the pericarp was cut or when extracted from a pericarp half. All statistics were performed in PAST v 4.15 [28].

## Results

**Fig 1:**
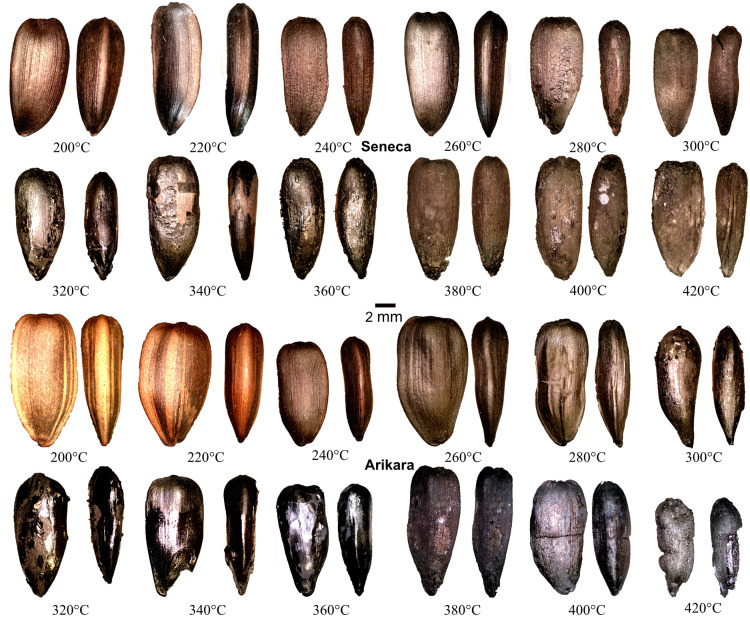
Examples of heated sunflower achenes with proximal ends at bottom. Each pair represents the same achene with planar views to the left and edge views to the right. For each image the original photograph was edited to remove the background. Brightness, contrast, and/or exposure were adjusted as needed to make features clear.

**Fig 2:**
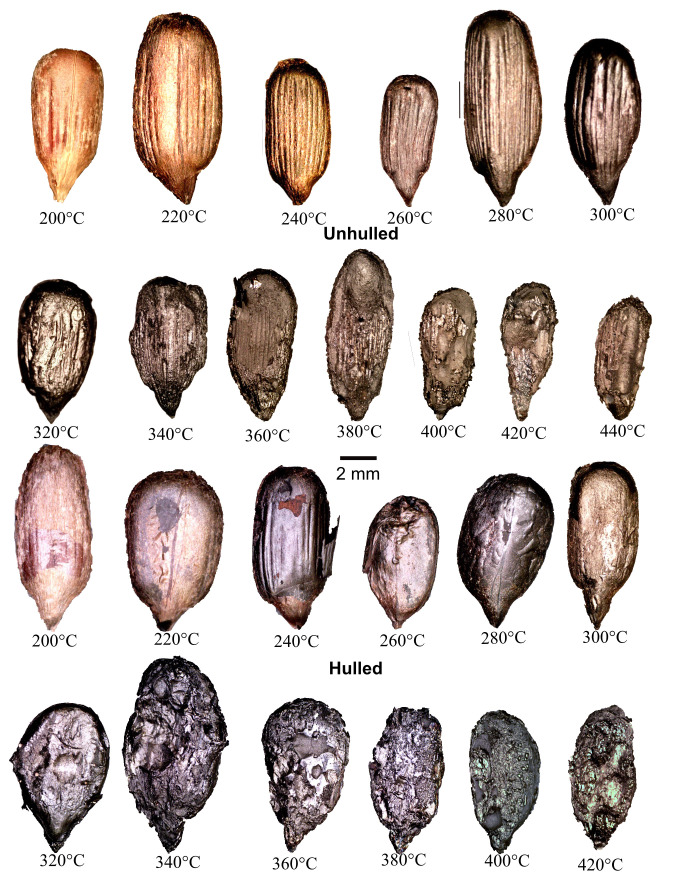
Examples of heated Arikara sunflower kernels with proximal ends at bottom. For each image the original photograph was edited to remove the background. Brightness, contrast, and/or exposure were adjusted as needed to make features clear.

**Fig 3:**
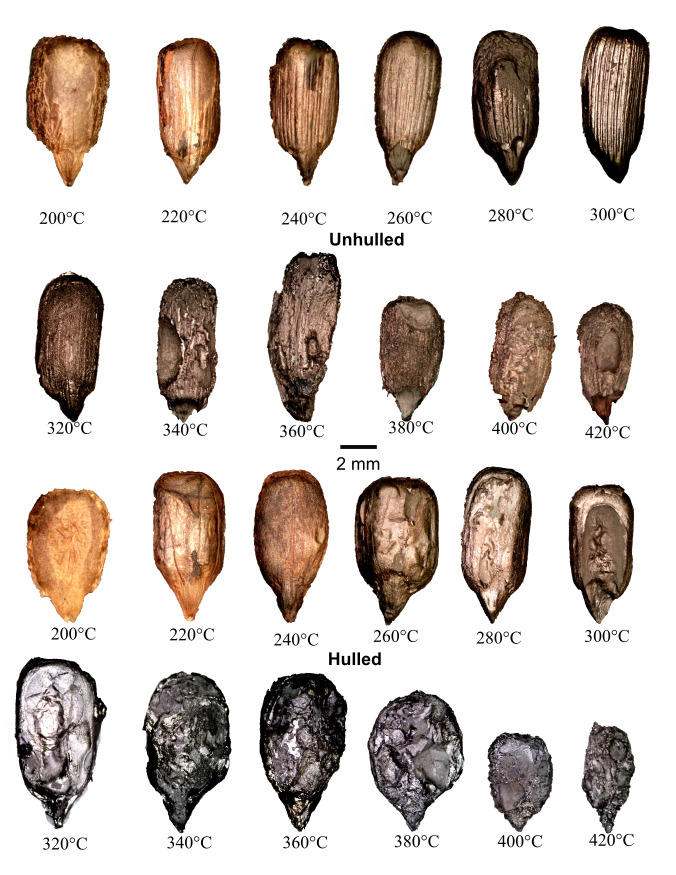
Examples of heated Seneca sunflower kernels with proximal ends at bottom. For each image the original photograph was edited to remove the background. Brightness, contrast, and/or exposure were adjusted as needed to make features clear.

All data are presented in S1 Table. Photographs of example achenes are presented for each temperature in Fig 1 and for kernels in Figs 2 and 3. Pericarps of achenes heated between 200 and 260°C did not fully blacken while those heated at temperatures above 260°C did. Surviving seed coats on hulled kernels were light brown at 200°C, while half of the kernels were black and the other half were brown. The cotyledons of hulled kernels heated at temperatures above 200°C were black. At 200°C unhulled kernel cotyledons were brown. At 220°C unhulled kernels were brown but had black interiors. At 240°C and above unhulled kernels were black throughout. Between 300 and 360°C most pericarp and kernel exterior surfaces were oily, and the interior surfaces of enclosing foil were sticky with burned oil. This is consistent with Braadbaart and associate’s [9, p. 322] finding for the evaporation of triglycerides between 340 and 370°C. At 310°C polysaccharides, lignin, and proteins were almost completely converted into aromatic moieties in achenes and kernels in Braadbaart and Wright’s [8, p. 1432] experiments. In the present experiments achenes and kernels heated at 380°C and above did not exhibit oily surfaces, nor was the burned oil on interior surfaces of the enclosing aluminum foil sticky. This is consistent with Braadbaart and associates’ [9, p. 322–323] finding that triglycerides had completely evaporated, and most lipid markers were absent at 370°C and none were present at 440°C.

**Fig 4:**
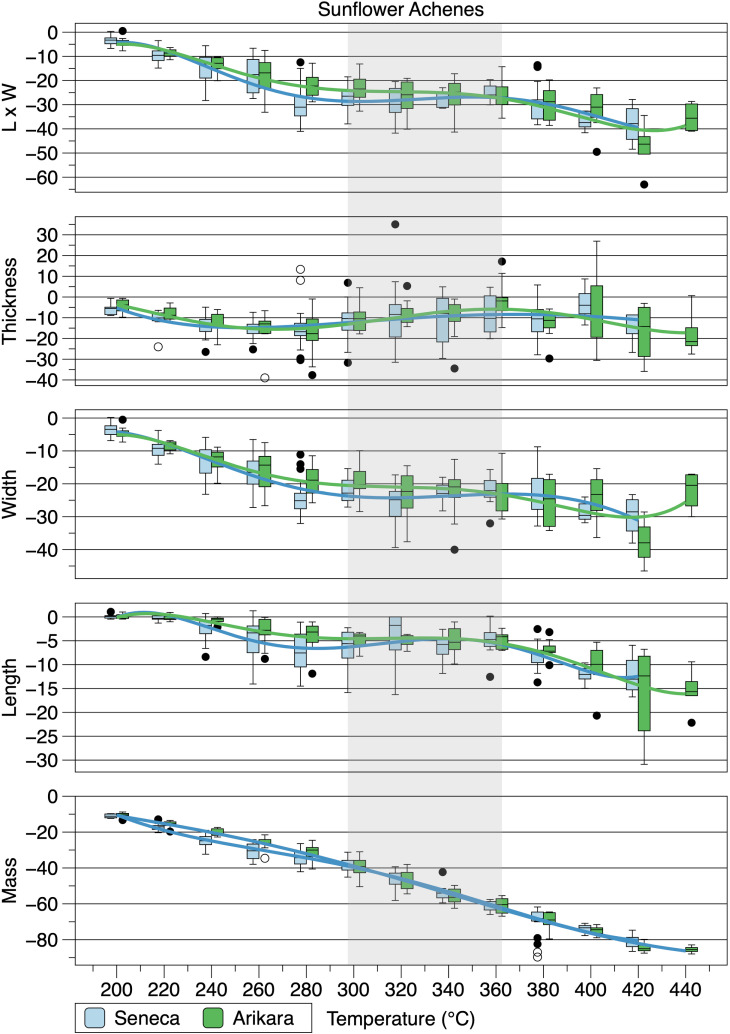
Box plots of percent changes of heated sunflower achenes from raw achenes. Gray shading is temperature range at which achene surfaces were oily after heating. The central line in each box is the median, the upper and lower edges ate the first and third quartile, respectively, the whiskers represent he minima and maxima, and dots are outliers (1.5 times greater than the difference between the first and third quartile. The connecting lines are fifth-order polynomial regressions used to illustrate trends.

Mean percent changes in achene metrics for each variety have high to very high, significant, negative Spearman rank order correlations with temperature except thickness, which has negligible, non-significant correlations (Table 1). All achene metrics for the two varieties except thickness have high to very high, positive, significant correlations with one another. Percent changes in achene mass were gradual (Table 1, Fig 4) exceeding 80 percent at 420°C consistent with Braadbaart and Wright’s [8] results. As shown in Fig 2, little change is evident in the various metrics between 300°C and 360°C, when achene exterior surfaces were oily after heating, following gradual decreases at lower temperatures. Metrics decreased again at temperatures above 360°C.

**Table 1:** Mean percent changes by temperature for achenes with Spearman rank order correlations (*ρ*).

°C	Arikara Mass	Seneca Mass	Arikara Length	Seneca Length	Arikara Width	Seneca Width	Arikara Thickness	Seneca Thickness	Arikara LxW	Seneca LxW
200	−10.47	−11.03	0.14	0.09	−4.48	−3.56	−4.04	−5.70	−4.33	−3.48
220	−15.69	−17.24	−0.21	−0.27	−8.46	−9.45	−8.09	−10.96	−8.65	−9.69
240	−20.45	−25.16	−0.71	−2.23	−12.93	−13.00	−12.84	−13.85	−13.55	−14.86
260	−26.26	−30.68	−3.11	−4.60	−15.63	−16.04	−15.65	−15.40	−18.12	−17.79
280	−30.87	−34.53	−3.85	−7.34	−18.98	−24.24	−16.58	−14.89	−22.09	−29.73
300	−39.73	−38.34	−4.82	−6.51	−19.31	−22.10	−9.50	−11.55	−23.14	−27.12
320	−47.21	−46.26	−4.91	−4.08	−22.52	−26.09	−8.83	−8.72	−26.32	−29.15
340	−56.59	−53.53	−5.05	−6.23	−23.71	−22.52	−9.31	−11.02	−27.54	−27.37
360	−60.88	−61.03	−5.03	−4.88	−22.20	−22.16	−1.33	−8.34	−26.14	−24.96
380	−69.37	−70.21	−6.72	−8.12	−25.63	−22.96	−12.73	−10.98	−29.66	−29.14
400	−74.89	−73.78	−10.45	−12.08	−23.99	−28.46	−6.42	−3.19	−32.23	−36.81
420	−84.44	−81.112	−15.75	−12.19	−37.74	−29.60	−16.85	−14.66	−47.30	−37.98
440	−85.46	--	−15.46	--	−22.00	--	−17.32	--	−35.20	--
Variety-Temperature *ρ*	−1.0000	−1.0000	−0.9890	−0.8601	−0.8626	−0.8866	−0.3022	−0.6701	−0.9780	−0.9048
Variety-Temperature *p*	0.0000	0.0000	0.0000	0.0003	0.0001	0.0001	0.3156	0.1375	0.0000	0.0000
Variety-Variety *ρ*	1.0000		0.8671		0.8741		0.9161		0.8671	
Variety-Variety *p*	0.0000		0.0003		0.00002		0.0000		0.0003	

The hulled and unhulled kernels of both varieties became increasingly degraded at temperatures above 300°C (see Figs 2 and 3). The unhulled cotyledon surfaces exhibited longitudinal ridges throughout the heating range, even at the highest temperatures when much of the cotyledons were consumed--ridges were visible on the remaining cotyledon surfaces (Figs 2 and 3). The ridges resulted from the kernels expanding against the interior surfaces of the pericarps. Hulled kernel cotyledon surfaces at temperatures ≤300°C were generally smooth apart from wrinkles in surviving seed coats. At 320°C and above hulled and unhulled kernels were generally partially consumed exhibiting voids/pits in cotyledon surfaces. After heating at 400°C two of the hulled Arikara kernels emerged in pieces as did seven of the hulled Arikara kernels heated at 420°C. Two of the hulled Seneca kernels were partially consumed at 420°C. The unhulled Arikara kernel from achenes heated at 440°C, while intact, were highly degraded and partially consumed (Fig 2).

**Fig 5:**
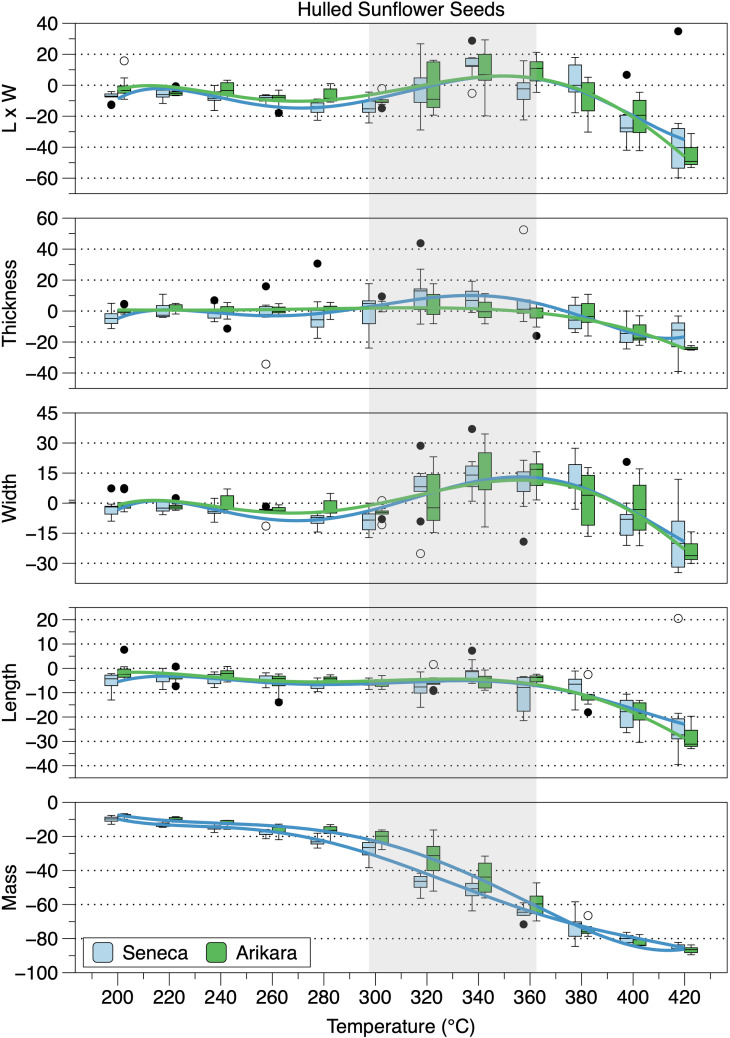
Box plots of percent changes of hulled sunflower kernels from raw kernels. Gray shading is temperature range at which kernel surfaces were oily after heating. See the Fig 4 caption for explanation of boxes and connecting lines.

**Fig 6:**
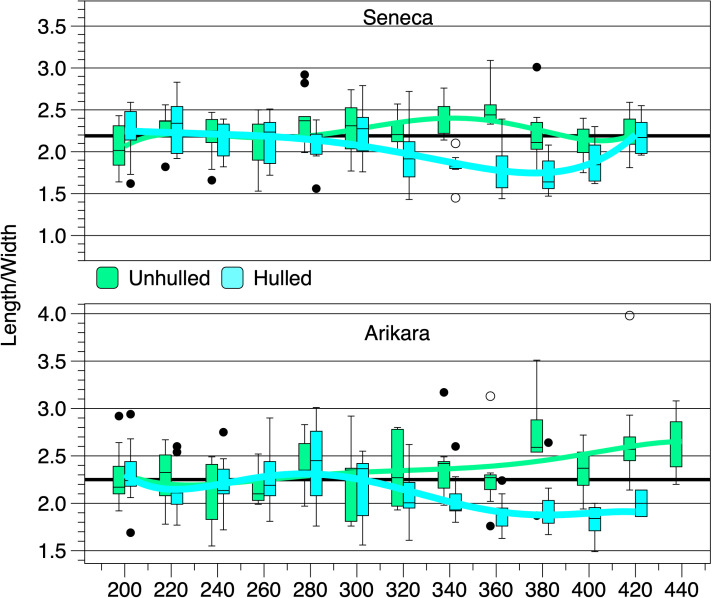
Box plots of heated kernel length:width ratios. Horizontal lines are the ratios for all unheated kernels of the respective varieties. See the Fig 4 caption for explanation of boxes and connecting lines.

There are very high, negative correlations between mean loss of mass and temperature for hulled kernels (Table 2, see Figs 4 and 5). There are high, negative correlations between mean decrease in length and temperature for hulled kernels. However, unlike achenes, mean widths of hulled kernels have negligible, non-significant, negative correlations with temperature. This is explained in large part by mean kernel widths increasing between 320 and 380°C before shrinking at 400 and 420°C, with greatest shrinkage occurring at 420°C. There are moderate to very high, positive correlations between the hulled kernels of the two varieties for the means of each metric except thickness, which has a moderate, positive, non-significant correlation. At temperatures lower than 320°C hulled and unhulled kernels have similar length:width ratios. However, mean width for unhulled kernels do not increase in the 320–380°C temperature range like hulled kernels. Rather, unhulled kernels become thinner as shown by length:width ratios (Fig 6, Table 3). The Arikara unhulled kernels’ highest ratio is at 380°C, while the Senecas’ is at 360°C. Unhulled Seneca kernel length:width ratios approach the mean value for all unheated heated kernels at 380–420°C, while Arikara unhulled kernel ratios remain high. This difference is reflected in the non-significant, correlation between length:width ratios for unhulled kernels of the two varieties (Table 4). There is a significant, strong, negative correlation for mean length:width ratio and temperature for the hulled Arikara kernels and a significant, moderate, negative correlation for hulled Seneca kernels. To the contrary, there is a significant, strong, positive correlation for unhulled Arikara kernels for length:width ratios and temperature, but a non-significant negligible correlation for Seneca unhulled kernels (Table 2). The percent changes in length:width ratios of the two varieties have similar patterns with little change at temperatures 200–300°C, above which there are substantial changes reflecting increased widths (Fig 6). Mean changes in length:width ratios of the two varieties have significant, strong, positive correlations (Table 3).

**Table 2:** Mean percent changes by temperature for hulled kernels with Spearman rank order correlations (*ρ*).

°C	Arikara Mass	Seneca Mass	Arikara Length	Seneca Length	Arikara Width	Seneca Width	Arikara Thickness	Seneca Thickness	Arikara LxW	Seneca LxW	Arikara L/W	Seneca L/W
200	−7.76	−9.96	−1.39	−5.22	−0.06	−2.32	0.39	−4.58	−1.37	−7.50	−1.02	−2.58
220	−9.83	−12.85	−3.36	−3.85	−1.63	−2.00	1.75	−0.07	−4.96	−5.79	−1.34	−1.85
240	−12.03	−14.9	−2.42	−4.48	−0.54	−3.47	−0.16	−1.05	−2.95	−7.76	−1.92	−0.87
260	−15.65	−17.85	−6.07	−4.73	−3.28	−3.98	0.51	−1.53	−9.14	−8.55	−3.20	−0.63
280	−16.77	−22.78	−4.42	−7.07	−2.42	−8.12	1.23	−2.61	−6.71	−14.58	−2.32	1.21
300	−20.68	−27.82	−5.51	−6.11	−4.81	−8.54	2.83	1.76	−10.06	−14.10	−2.60	2.99
320	−33.47	−46.76	−5.63	−7.61	3.22	6.05	3.28	12.01	−2.48	−1.91	−7.86	−11.23
340	−43.89	−51.19	−4.97	−1.26	12.93	14.52	0.35	7.42	7.50	12.92	−13.77	−13.11
360	−59.28	−64.49	−4.37	−10.40	14.74	9.23	−2.45	7.47	9.75	−3.29	−16.77	−15.60
380	−75.12	−71.77	−11.38	−7.62	1.27	11.79	−2.28	−4.00	−8.15	2.84	−12.38	−17.83
400	−82.58	−80.08	−18.91	−18.15	−2.46	−7.48	−13.98	−14.15	−20.78	−24.07	−15.82	−10.62
420	−86.72	−85.22	−27.96	−22.57	−23.53	−18.085	−24.00	−16.59	−44.51	−33.58	−4.12	−4.53
Temperature-Variety *ρ*	−0.9582	−0.9787	−0.7972	−0.7483	−0.0345	0.0428	−0.6224	−0.2945	−0.4568	−0.2673	−0.8392	−0.5804
Temperature-Variety *p*	0.0000	0.0000	0.0019	0.0051	0.9141	0.8950	0.0307	0.3528	0.1355	0.4009	0.0006	0.0478
Variety-Variety *ρ*	1.0000		0.6154		0.9161		0.5525		0.7483		0.7063	
Variety-Variety *p*	0.0000		0.0332		0.0000		0.0625		0.0051		0.0102	

**Table 3:** Mean Length/Width ratios of hulled and unhulled sunflower kernels by temperature with Spearman rank order correlations (*ρ*).

°C	Arikara		Seneca	
	Hulled	Unhulled	Hulled	Unhulled
200	2.37	2.28	2.22	2.05
220	2.06	2.28	2.31	2.29
240	2.24	2.10	2.16	2.19
260	2.20	2.17	2.16	2.08
280	2.41	2.46	2.09	2.37
300	2.31	2.24	2.23	2.29
320	2.05	2.34	1.94	2.24
340	2.04	2.40	1.83	2.41
360	1.91	2.29	1.80	2.56
380	1.75	2.68	1.74	2.21
400	1.80	2.36	1.88	2.12
420	2.00	2.69	2.19	2.22
440	--	2.63	--	--
Temperature-Variety *ρ*	−0.7422	0.7565	−0.5990	0.2242
Temperature-Variety *p*	0.0057	0.0027	0.03960	0.4837
Hulled-Unhulled *ρ*	−0.4709		−0.2351	
Hulled-Unhulled *p*	0.1223		0.4620	

**Table 4:** Spearman rank order correlation (*ρ*) analyses of Arikara and Seneca sunflower Mean Length/Width ratios of hulled and unhulled kernels by temperature.

°C	Hulled		Unhulled	
	Arikara	Seneca	Arikara	Seneca
200	2.37	2.22	2.28	2.05
220	2.06	2.31	2.28	2.29
240	2.24	2.16	2.10	2.19
260	2.20	2.16	2.17	2.08
280	2.41	2.09	2.46	2.37
300	2.31	2.23	2.24	2.29
320	2.05	1.94	2.34	2.24
340	2.04	1.83	2.40	2.41
360	1.91	1.80	2.29	2.56
380	1.75	1.74	2.68	2.21
400	1.80	1.88	2.36	2.12
420	2.00	2.19	2.69	2.22
Hulled-Unhulled *ρ*	0.7418		0.1539	
Hulled-Unhulled *p*	0.0037		0.4863	

**Fig 7:**
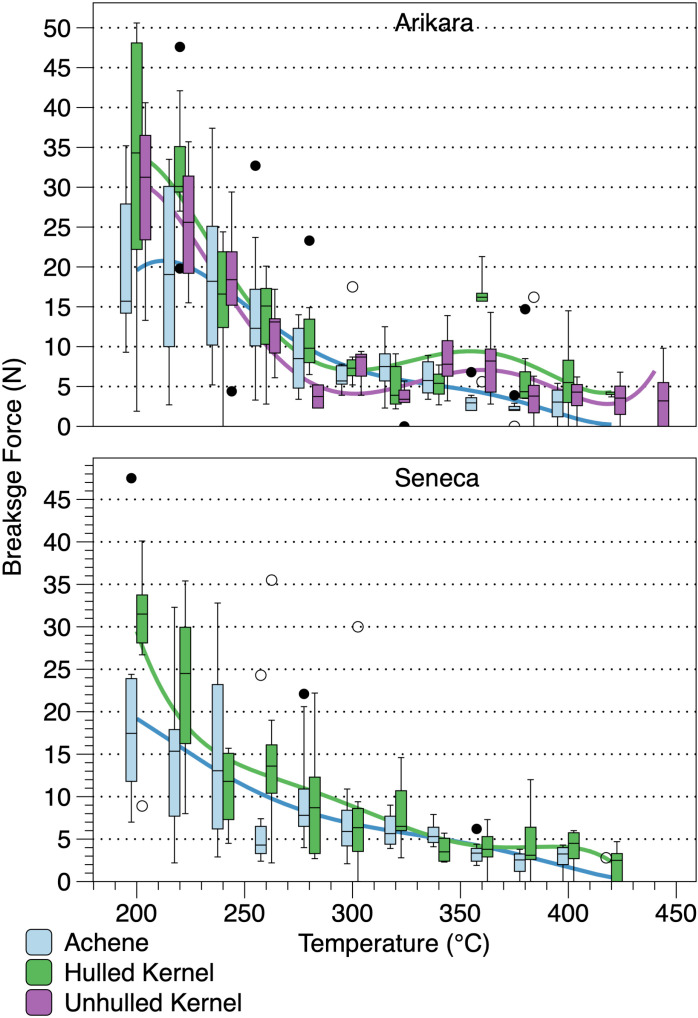
Box plots of breakage force (N) for heated sunflower achenes and kernels. See the Fig 4 caption for explanation of boxes and connecting lines.

Mean breakage force and mean percent change in mass of achenes have very strong, positive Spearman rank order correlations (Arikara: *ρ* = 0.9860, *p* = 0.0000; Seneca: *ρ* = 0.9930, *p* = 0.0000) indicating that it is loss of mass from mobilization of lipids, lignin, and proteins [9] that determines achene compression strength. Achene compression strength decreases gradually, with mean N reaching ~0 at 420°C. The pericarps of the Arikara achenes heated at 440°C (Fig 7) emerged in pieces (n = 2), mostly ash (n = 7), or were completely consumed (n = 1) leaving only the charred seed. Breakage force for Arikara hulled kernels has a significant strong, negative correlation with temperature, while Seneca hulled kernels have a significant very strong, negative correlation (Table 5). Of note, however, is that eight of the Arikara kernels heated at 340°C and five heated at 360°C compressed without breaking. Breakage of four of the remaining kernels heated at 360°C required an average force of 15.1 N, substantially higher than average force for kernels heated at 380°C (5.96 N). Six of the Seneca kernels heated at 360°C compressed without breaking, although breakage force for the remaining kernels was not high like that for the Arikara kernels. Breakage force for unhulled Arikara kernels has a significant, moderate, negative correlated with temperature (Table 5). Unhulled Arikara kernels heated at 360°C, unlike hulled kernels, did not compress without breaking. Unhulled Seneca kernels mostly broke or split into cotyledons when extracted from the enclosing pericarps. While sufficient numbers of Seneca kernels could be measured by refitting the cotyledons together, the prior breakage and splitting precluded breakage force testing. Unhulled Arikara kernel mean breakage force has very strong correlations with mean breakage force for hulled kernels of both varieties (Table 5).

**Table 5:** Mean breakage force (N) for sunflower achenes and kernels and Spearman rank order correlations (*ρ*). Unhulled Arikara kernels are compared with hulled kernels of both varieties (Arikara left cells, Seneca right cells).

	Achenes		Hulled Kernels		Unhulled Arikara Kernels Force	
°C	Arikara Force	Seneca Force	Arikara Force	Seneca Force		
200	19.91	19.18	32.97	29.17	30.93	
220	19.26	15.34	32.83	22.63	24.62	
240	18.57	14.91	16.13	10.88	19.15	
260	14.02	10.59	12.97	14.62	13.25	
280	8.64	10.71	11.54	9.91	3.37	
300	6.04	6.17	8.34	8.38	6.60	
320	7.33	5.99	5.10	8.02	3.46	
340	5.73	5.72	5.27	3.75	7.12	
360	3.24	3.40	15.10	3.81	7.74	
380	2.14	2.23	5.96	4.35	5.90	
400	2.88	2.80	6.13	3.99	2.91	
420	0.00	0.28	4.1	2.3	3.64	
440	0.00	--	--	--	5.20	
Temperature-Variety *ρ*	−0.9876	−0.9700	−0.7972	−0.9301	−0.6868	
Temperature-Variety *p*	0.0000	0.0000	0.0002	0.0000	0.0095	
Variety-Variety *ρ*	0.98601		0.7832		0.9277	0.9059
Variety-Variety *p*	0.00000		0.0026		0.0000	0.0000

Results of Mann-Whitney tests for breakage force equal medians between sunflower achenes and hulled kernels with common bean seed cotyledons and squash seeds using data from previous experiments using the same conditions and equipment [21,22] are presented in Table 6. In these tests I combined sunflower achenes and hulled kernels from both varieties, respectively, all common bean seed cotyledons from both varieties [21], and *C. pepo* squash [22]. Median breakage force for common bean cotyledons were significantly greater than sunflower achenes and hulled kernels at all temperatures. Differences in medians between sunflower achene and squash seed breakage force are not significantly different below 300°C. At 300, 320, and 340°C sunflower achenes have significantly greater medians, while at 360 and 380°C they are not significantly different. There is no significant difference between breakage force medians for hulled sunflower kernels and squash seeds below 280°C, while at 280°C and above hulled sunflower kernels have greater medians except at 380°C where there is no significant difference.

**Table 6:** Mann-Whitney tests of Breakage force for Sunflower achenes and hulled kernels and common bean cotyledons and squash seeds.

°C	*U*	*z*	*p* ^a^	Mean Rank	
				Sunflower Achenes	Bean Seed Cotyledons
200	20.0	4.7628	0.0001	5.3846	14.6150
220	10.0	5.1260	0.0001	5.5000	15.0000
240	16.0	4.7802	0.0001	4.9211	14.5790
260	16.0	4.9639	0.0001	5.6500	14.8500
280	11.5	4.8327	0.0001	5.4459	13.5540
300	2.0	5.1975	0.0001	5.0526	14.4470
320	37.5	4.3827	0.0001	6.1875	14.3130
340	8.0	4.8498	0.0001	5.5000	13.0000
360	14.0	4.7589	0.0001	5.0000	14.0000
380	10.5	4.0330	0.0001	3.0517	11.9480
400	22.0	3.8831	0.0002	3.5313	12.9690
°C	*U*	*z*	*p* ^a^	Sunflower Achenes	Squash Seeds
200	83.0	2.2684	0.0219	7.8000	10.2000
220	155.0	1.2038	0.2306	9.1250	11.3750
240	171.0	0.2486	0.7998	9.0000	10.5000
260	158.0	0.6287	0.5228	9.6842	9.8158
280	159.0	0.6135	0.5396	10.3160	9.1842
300	23.5	4.3748	0.0001	13.5970	4.9028
320	86.5	2.3254	0.0189	12.3190	6.1806
340	64.5	3.3774	0.0006	12.8030	6.6974
360	133.0	0.9050	0.3545	10.0560	8.4444
380	55.0	0.9819	0.3244	6.9583	5.5417
°C	*U*	*z*	*p* ^a^	Sunflower Hulled Kernels	Bean Seed Cotyledons
200	25.0	4.2819	0.0001	4.4722	14.0280
220	24.5	3.6982	0.0002	3.2031	13.2970
240	2.0	4.9167	0.0001	3.4857	14.5140
260	22.0	4.6051	0.0001	5.0789	14.4210
280	18.0	4.5407	0.0001	5.2500	13.2500
300	19.0	4.4286	0.0001	4.4286	13.5710
320	44.5	4.0764	0.0001	6.0128	13.9870
360	78.0	1.9860	0.0447	5.5455	11.4550
380	106.0	1.5356	0.1271	7.9143	10.0860
400	75.5	2.0773	0.0355	5.4697	11.5300
°C	*U*	*z*	*p* ^a^	Sunflower Hulled Kernels	Squash Seeds
200	116.0	0.4334	0.6706	8.6250	7.8750
220	64.0	2.1605	0.0265	7.9375	8.5625
240	129.0	0.6837	0.4949	7.1143	10.8660
260	112.5	1.5506	0.1199	10.6250	7.8750
280	80.0	2.7512	0.0049	11.7030	7.2973
300	22.0	4.1178	0.0001	11.6970	5.3030
320	112.5	1.2926	0.1985	10.9000	7.1000
360	62.5	2.4067	0.0155	9.2031	7.2969
380	30.5	3.2820	0.0005	11.8830	3.6167

^a^9999 permutation Montecarlo *p* -values

## Discussion and conclusions

Sunflower is one of two crops in the indigenous Eastern Agricultural Complex of North America that contributes to current global agricultural production. Sunflower achenes and kernels recovered from open-air eastern North America indigenous archaeological sites are generally carbonized. Charring can result in changes to achene and seed sizes. Because size is the primary criterion by which paleoethnobotanist distinguish achenes of domesticated (length ≥7 mm, length x width product >23) from wild (smaller) plants, several experiments have been carried out since the 1950s to understand how achene and seed size changes during carbonization. These experiments have typically focused on shrinkage in achene and seed lengths and widths. Here, I have related the results of a series of systematic heating experiments of sunflower achenes and kernels to arrive at better understandings of the morphological changes caused by exposure to high temperatures in oxygen-limited contexts and to assess compression strength as a proxy for survival in the archaeological record.

On the basis of their heating experiments for 60 minutes in anaerobic conditions, Braadbaart and Wright [8,9] suggested various temperature ranges at which sunflower achenes were likely char to and survive in the archaeological record. The widest temperature range suggested was 310°C to 600°C [8], while Wright [12] suggested a range of 370°C to 500°C. Braadbaart and Wright [8,9] indicate that at temperatures below 310°C proteins and polysaccharides survive in achenes resulting in soil microbial attack, lessening chances for preservation, while oils evaporate at 340°C and are completely absent at 370°C. In the current experiments achenes heated at 380°C did not exhibit the oily surfaces that were present on achenes heated between 300°C and 360°C. If it is accepted that the evaporation of oils is necessary to enhance the potential of archaeological preservation, then the potential range of achene preservation is very narrow based on the current experiments because breakage force reaches ~ 0N by 420°C; suggesting that under oxygen-limited conditions achenes heated at 380°C to <420°C are likely to survive. On the other hand, it is feasible that when oil is extruded from achenes at temperatures ≥300°C and proteins, lignin, and polysaccharides are transformed into aromatic moieties, any microbial attack would be on the labile oils, not the recalcitrant aromatic portions of the achenes.

Of importance in considering microbial attack is the effect of heat on soil microbiota. Dry soil heated to as low as 80°C will kill up to 99% of soil fungi, while temperatures of 90°C to 120°C will kill 99% of soil bacteria; 10°C to 20°C lower temperatures in moist soil have the same effects [29–31]. Soil temperatures under open hearths regularly exceed 120°C [25,27,32], likely sterilizing the soil below and immediately surrounding hearths though repeated heating. This suggests that sunflower achenes and kernels charred as a result of exposure to heating by hearth fires would not be subject to microbial attack in their primary depositional contexts. Periodic cleaning of hearths would remove charred sunflower achenes and kernels along with ash, wood charcoal, and any sterilized soil scraped up with the fire debris. Dumping in abandoned storage pits that themselves had been fired or middens with high concentrations of wood ash would be detrimental to soil microbes [33]. It is possible, then that achenes and kernels charred at 300–380°C can preserve in the archaeological record as suggested by the lower portion of Braadbaart’s and Wright’s [8] range. It is notable that this temperature range is higher than the range at which maize kernels and common bean seeds that are found in the same context as maize kernels are likely to have charred (200°C to 250/60°C) [21]. Compression testing suggest that sunflower achenes and hulled kernels are less likely to preserve intact than common bean cotyledons at all tested temperatures, having significantly lower breakage force medians. Temperature at and above 280°C sunflower achenes and hulled kernels are more likely to preserve intact than are squash seeds (although the differences in medians are not statistically different for achenes at 360 and 380°C).

The greatest difference in metrics of the current experiments from those of previously reported experiments is the change in hulled kernel widths heated between 320°C and 380°C. In the current experiments mean widths increase by over 10 percent in some cases. This contrasts with unhulled kernels in the same temperature range. Length:width ratios were≤2.05 for hulled kernels of both varieties, whereas unhulled Arikara kernel ratios were >2.20. Coupled with the longitudinal ridges present on unhulled kernels it is possible that this ratio may be used to infer whether kernels recovered from the archaeological record were hulled or not prior to charring.

**Fig 8:**
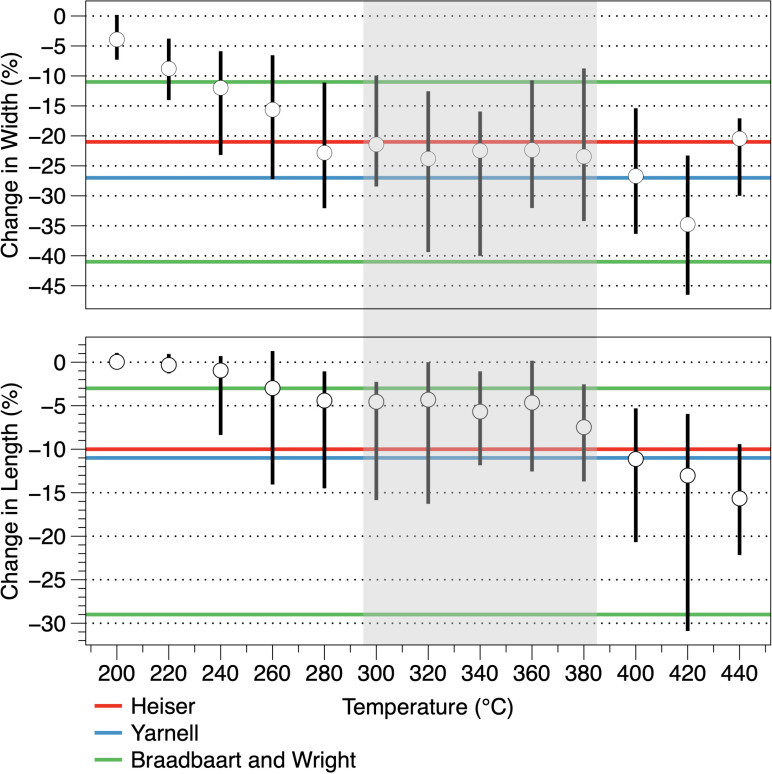
Mean change in heated achene length and width. Whiskers are the minimum and maximum ranges. Previously published correction factors used to estimate original achene dimensions from charred achenes recovered from archaeological sites are shown as horizontal lines. Shading indicates temperature range most likely to result in achenes preserving in the archaeological record (see text for explanation).

If achenes recovered from archaeological contexts are likely to have charred in the temperature range of 300–380°C applying previously published correction factors to obtain original achene [13] size would be inaccurate (Table 7; S2 Table), resulting in type 1 and type 2 errors in determining domestication status. These results amplify Braadbaart’s and Wright’s [8, pp. 151–153] critique of using a single correction factor to estimate original achene size. Results of the present experiments go farther in questioning whether it is feasible to estimate original achene size by applying a correction factor or a range of factors to charred achenes. As shown in Fig 8 suggested correction factors overestimate shrinkage of achenes in the current experiments at all but a small range of temperatures in the range suggested by Braadbaart and Wright [8].

**Table 7:** Differences between length and width (mm) of preheated achenes and application of correction factors to achenes after charring. Paired *t*-tests indicate significant differences (*p* < 0.05) between the original metrics and those estimated from the achenes after charring except for those marked *. Data used in the *t*-*t*ests are provided in S2 Table.

	Temperature	Heiser		Yarnell achene		Yarnell kernel		Braadbart & Wright Low		Braadbart & Wright High	
		Mean	Range	Mean	Range	Mean	Range	Mean	Range	Mean	Range
Length	300	−0.41	−0.81 - 0.90	0.53	−0.72 - 1.02	−1.06	−2.93–0.33	−0.35	−0.45 - 0.08	2.47	0.93 - 3.16
	320	0.61	−0.91 - 1.25	0.72	−0.82 - 1.37	−1.72	−3.81–0.25	−0.18*	−1.59 - 0.37	2.76	0.92 - 3.62
	340	0.44	−0.32 - 0.96	0.56	−0.23 - 1.08	−0.67	−3.73–1.81	−0.34	−0.96 - 0.18	2.56	1.43 - 3.28
	360	0.51	−0.42 - 1.17	0.61	−0.33 - 1.29	−1.49	−4.96–0.01	−0.24	−1.10 - 0.37	2.54	1.42 - 3.37
	380	0.19	−0.55 - 0.74	0.29	−0.46 - 0.84	−1.94	−3.70 - −0.57	−0.56	−1.21 - 0.04	2.23	1.23 - 2.85
	300-380	0.44	−0.91–1.25	0.55	−0.82–1.37	−1.37	−4.96–1.81	−0.33	−1.59–0.37	2.52	0.92–3.62
Width	300	−0.44	−2.12 - 0.83	−0.18*	−1.90 - 1.12	−0.53	−1.86 - 1.08	−0.87	−2.49 - −0.35	0.42	−1.38 - 1.88
	320	−0.47	−1.62 - 0.28	−0.20*	−1.37 - 0.60	0.02	−1.71 - 2.38	−0.93	−2.03 - −0.12	0.43	−0.79 - 1.44
	340	−0.42	−1.53 - 0.11	−0.13*	−1.33 - 0.45	0.60	−0.83 - 2.69	−0.90	−1.86 - −0.44	0.54	−0.86 - 1.22
	360	−0.32	−0.88 - 0.48	−0.05*	−0.67 - 0.80	0.17*	−1.50 - 1.74	−0.77	−1.33 - −0.05	0.58	−0.20–1.55
	380	−0.44	−1.85 - 1.24	−0.17*	−1.61 - 1.53	0.22*	−1.29–1.72	−0.90	−2.25 - 0.75	0.47	−1.06–2.21
	300-380	−0.42	−2.12–1.24	−0.15	−1.90–1.53	0.80	−1.86–2.69	−0.88	−2.49–0.75	0.48	−1.38–2.21
LxW	300	−0.63	−10.21–12.09	3.32	−7.35–19.28	−11.88	−32.69–13.16	−10.06	−18.89 - −0.46	24.52	15.36–44.56
	320	−3.46	−18.28–6.31	0.44	−14.05–12.34	−9.82	−30.90–9.19	−12.77	−28.38 - −3.11	24.20	17.60–37.39
	340	−2.58	−15.28–7.37	1.61*	−12.06–12.13	2.97*	−23.35–37.20	−12.57	−22.96 - −3.98	25.97	19.21–31.76
	360	−0.60*	−8.89–8.65	3.27	−5.73–13.47	−7.69	−36.72–21.24	−9.83	−16.43 - −1.29	23.98	19.60–32.71
	380	−4.52	−14.42–8.65	−0.81*	−10.51–12.84	−9.05	−25.40–13.31	−13.36	−23.76 - −0.62	22.98	17.68–33.08
	300-380	−2.37	−18.28–12.09	1.55	−14.05–19.28	−7.23	−36.72–37.20	−11.73	−28.38–3.98	24.32	19.60 - 44.56

As shown in Table 7, the application of previously published correction factors to charred achenes in the temperature range of 300–380°C resulted in substantial under and over estimations at the range extremes. Paired *t*-tests indicate that with one exception estimated length and original length means are significantly different. Estimated width means are significantly different from original mean widths for all but Yarnell’s [13] correction factor for achenes and his correction factor to estimate achene width from charred kernel widths at 360 and 380°C. While Braadbaart’s and Wright’s [8] range captured the original achene side in most instances, there are large over and underestimates in the for both length and width that might lead to erroneous conclusions about individual achenes or achene assemblages. It is unlikely, however that sunflower achenes recovered from a site had been all charred at the same temperature. In all cases paired t-tests for the full range of 300–380°C indicate significant differences between original and estimated metrics. Lentz and associates [34] suggested that achene length x width products >23 indicate domesticated plants based on measurements of wild sunflower achenes by Heiser [35]. As expected, based on length and width estimates for uncharred achenes, differences between original and estimated length x width products have wide ranges and in only two cases do paired *t*-*t*ests indicate the means of original and estimated products are not significantly different (Table 7).

As previously established charred sunflower achenes and seed dimensions change as a result of charring. For the most part these changes are decreases on length and width. The present experiments resulted in achene length and width shrinkage throughout the heating range and mean length and width shrinkage was strongly to very strongly correlated with temperature. However, this did not hold for shelled kernels. While mean length decreased throughout the heating range and is very strongly correlated with temperature, mean widths increased in the 320–380°C range. Constrained by the pericarp, unhulled kernels were narrower than the hulled kernels but exhibited longitudinal ridges that were not present on the hulled kernels. This suggests that is possible to determine if charred kernels recovered from an archaeological site were hulled prior to exposure to heat. Achens and kernels exhibited increased degradation at temperatures above 300°C with kernels exhibiting pits and voids.

Mean compression strength decreased with and is very strongly correlated increased temperature for achenes and hulled kernels. Achenes have little or no compression strength when heated at the upper heating range of the present experiments. Sunflower achenes and hulled kernels exhibit lower compression strength at all temperatures than dried common bean seeds potentially indicating lower potential to preserve in the archaeological record. Sunflower achenes and squash seeds differed little in compression strength, while mean hulled kernels compression strength was generally greater than squash seeds above 260°C, suggesting they have a greater potential for preservation in the archaeological record.

Several correction factors have been offered to estimate the original dimensions of sunflower achenes from charred achenes or kernels. There is great potential that application of correction factors with substantially under or overestimate original achene dimensions. As a result, their application is likely to result in type 1 and type 2 assignment errors for determining if a carbonized achene or kernel originated from a domesticated plant. Of greater potential is Smith’s [2] suggestion that charred achenes and kernels from archaeological sites be compared to those of charred contemporary wild kernels. However, given that his experiments involve a single temperature (400°C), there is need to perform additional heating experiments at a range of temperatures given that 400°C is at the upper range of temperatures where pericarps survive heating in oxygen limited contexts. Based on Braadbaart and associates’ [8,9] analyses and results of the current experiments, a range of 320–380°C seems appropriate.

## Supporting information

S1 Table Sunflower achene and kernel metrics. (XLSX)

S2 Table Application of correction factors to experimentally charred sunflower achenes and kernels. (XLSX)
